# Impact of Vitamin D Supplementation on the Clinical Outcomes and Epigenetic Markers in Patients with Acute Coronary Syndrome

**DOI:** 10.3390/ph16020262

**Published:** 2023-02-09

**Authors:** Neven Sarhan, Ahmed Essam Abou Warda, Saud Alsahali, Abdalla Salah Alanazi

**Affiliations:** 1 Clinical Pharmacy Department, Faculty of Pharmacy, Misr International University, Cairo 11828, Egypt; 2 Clinical Pharmacy Department, Faculty of Pharmacy, October 6 University, Giza 12585, Egypt; 3 Department of Pharmacy Practice, Unaizah College of Pharmacy, Qassim University, Qassim 6688, Saudi Arabia; 4 Department of Clinical Pharmacy, College of Pharmacy, Jouf University, Sakaka 72388, Saudi Arabia; 5 Health Sciences Research Unit, Jouf University, Sakaka 72388, Saudi Arabia

**Keywords:** acute coronary syndrome, vitamin D receptor, pharmacogenetics, cardiac fibrosis, non-coding RNA

## Abstract

Vitamin D has recently been found to influence the renin-angiotensin system (RAS); it can reduce the effects of renin-angiotensin system inhibitors (RASI) by decreasing plasma renin. This study examines the effect of vitamin D supplements on cardiac fibrosis markers, echocardiographic parameters, and epigenetic markers in patients with established acute coronary syndrome (ACS). It also looks at the incidence of vitamin D receptor (VDR) gene polymorphisms *Apa I (rs7975232), Bsm I (rs1544410), Taq I (rs731236),* and *Fok I (rs2228570)* and its association with the development of secondary major acute cardiovascular events (MACE) and heart failure (HF). A randomized controlled trial in which patients were divided into two groups was performed. Group 1 comprised of 125 ACS patients who received ACS standard therapy alone, while Group 2 consisted of 125 ACS patients who received ACS standard therapy plus vitamin D according to their vitamin D levels. Patients were monitored for 24 months to find subsequent MACE and HF. Vitamin D therapy for ACS patients resulted in a substantial decline in end systolic and end diastolic volumes (*p* = 0.0075 and 0.002, respectively), procollagen type III N-terminal peptide (PIIINP) and soluble ST2 levels (*p* = 0.007 and 0.001, respectively), as well as in ejection fraction and vitamin D level (*p* = 0.0001 and 0.008, respectively). In addition, vitamin D treatment was linked to a significant decline in the levels of noncoding RNA, such as mir361, lncRNA MEG3, and lncRNA Chaer (*p* = 2.9 × 10^−4^, 2.2 × 10^−6^, and 1.2 × 10^−5^, respectively). Furthermore, patients who suffered MACE had significantly higher levels of the *Bsm I CC* and *Fok I GG* genotypes (*p* = 4.8 × 10^−4^ and 0.003, respectively), while patients with HF had significantly higher levels of the *Taq I AA* genotype (*p* = 4.2 × 10^−7^). Supplementing ACS patients with vitamin D has been demonstrated to limit cardiac fibrosis and echocardiographic parameters, as well as epigenetic markers. Additionally, MACE and HF among ACS patients may be related to genetic variations among VDR gene polymorphisms.

## 1. Introduction

Acute coronary syndrome (ACS) is a collection of ailments that include unstable angina, ST-elevation myocardial infarction (STEMI), and non-ST elevation myocardial infarction [1]. It is a subtype of coronary heart disease (CHD), the most prominent form of heart disease in individuals over the age of 35 and the cause of one-third of all deaths in this age group [2]. About 9.1 million fatalities worldwide are attributable to CHD each year, per the American Heart Association’s 2021 statistics report [3].

**Figure 1:**
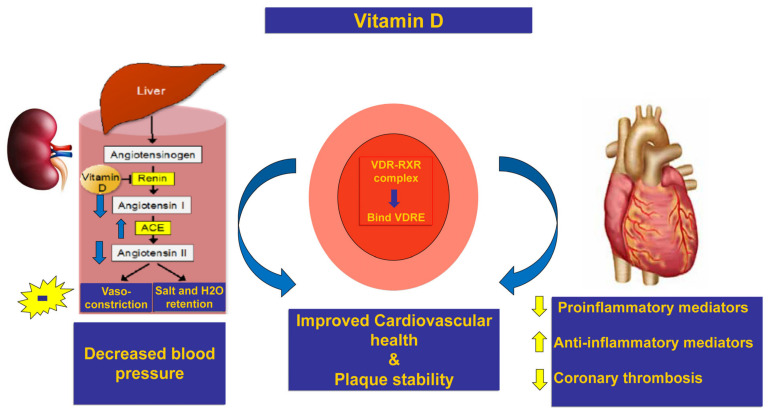
Possible mechanisms by which vitamin D may have additional protective effects when used in conjunction with other treatments for ACS patients through inhibition of inflammatory state, increased ACE as well as its antithrombotic and cardioprotective effects. ACE—angiotensin-converting enzyme; ACS—Acute coronary syndrome; VRD—vitamin D receptor; RXR—retinoid X receptor; and VDRE—Vitamin D response element. Arrows pointing up and down denote an increase and a decline, respectively; (-) symbol indicates inhibition.

Vitamin D receptor (VDR) and 1-hydroxylase, the enzyme responsible for converting inactive vitamin D to its active form, are expressed by endothelium, fibroblasts, heart muscle, and smooth muscle. Activation of VDR by calcitriol or its derivatives can decrease angiotensin I release and localized angiotensin II production in cardiac, kidney artery, and renal tissues as shown in Figure 1. Furthermore, it has been shown that vitamin D enhances the production of the enzyme ACE 2, which is responsible for converting excess amounts of angiotensin II into angiotensin 1–7 [4]. Consequently, angiotensin 1–7’s beneficial effects against hypertension, inflammation, and fibrosis are enhanced. The anti-inflammatory, antiproliferative, antifibrotic, anti-diabetic, and antithrombotic implications of vitamin D have been demonstrated in several human studies to serve a crucial role in the prevention and treatment of major CVDs [5,6].

Vitamin D has recently been hypothesized to be capable of controlling the renin-angiotensin system (RAS) by lowering plasmatic levels of renin and angiotensin, the expression of ACE 2 and angiotensin receptor 1, and suppressing plasma renin activity. As a corollary, vitamin D may interfere with the effects of renin-angiotensin system inhibitors (RASI) (Figure 1) [7,8]. The VDR gene is found on chromosome 12’s long arm and contains 11 exons (12 q12-14). Polymorphisms in the VDR gene may modify VDR gene expression and mRNA stability, which in turn may affect VDR gene function and vitamin D action [9,10].

Long noncoding RNA (lncRNA) is commonly used to refer to transcripts longer than 200 nucleotides that are incapable of encoding proteins. Understanding the functions of lncRNA in human disease has revealed new molecular insights and will result in innovative therapeutic and diagnostic methods [11]. Diagnostic value of circulating long noncoding RNAs (lncRNAs) in heart diseases has never been studied, even though multiple trials have scrutinized short RNAs such as microRNAs as predictive markers for cardiovascular diseases (albeit with relatively small numbers of patients, with a very few exceptions) [12]. A lot of evidence points to non-coding RNAs as critical regulators in the onset and progression of cardiovascular events, but their roles in these processes are still poorly understood [13].

Few studies have attempted to examine the correlation between RASI and vitamin D levels, and there is no information about individuals with ACS who have had their vitamin D levels measured, or their levels of non-coding RNA, which could be used as prognostic indicators for ACS-related problems [7,8,14] Therefore, the purpose of this investigation is to examine the link between both vitamin D levels and indeed the occurrence of HF and other major adverse cardiovascular events (MACE). Additionally, the efficacy of vitamin D supplements on biomarkers of cardiac fibrosis, such as Procollagen type III N-terminal peptide (PIIINP) and soluble ST2, as well as echocardiographic parameters and epigenetic markers, such as micro- and lncRNAs. In addition, we investigated non-coding RNAs as a prospective serum biomarker for the occurrence of HF and MACE. The study’s secondary objectives are to evaluate the prevalence of VDR gene polymorphism in Egyptian patients with ACS, and to determine whether this polymorphism was linked to the development of HF or MACE.

## 2. Results

**Figure 2:**
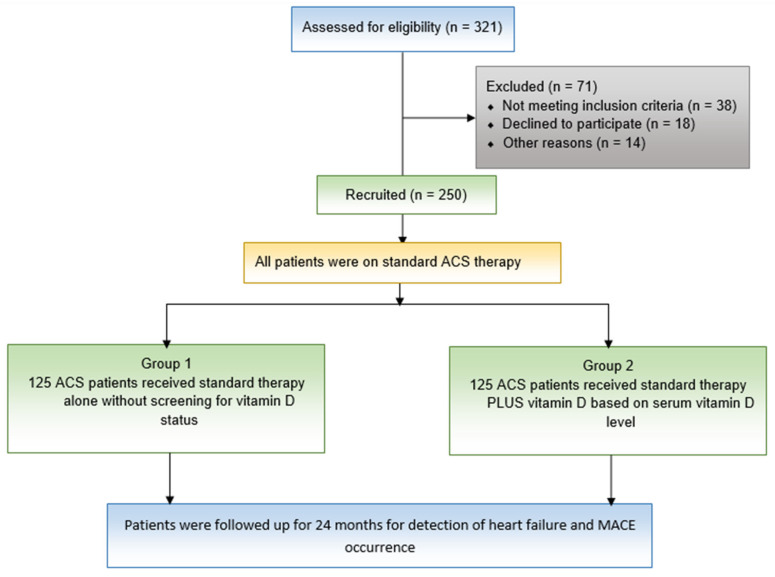
Flow chart show studied patients’ recruitment in the study.

Of the 321 adult ACS patients who participated in the current trial, 70 did not qualify for inclusion due to study-specific exclusion criteria. After recruiting 250 patients, they were divided into two groups, Group 1 (*n* = 125) and Group 2 (*n* = 125) as shown in Figure 2, their baseline characteristics shown in Table 1. There was no statistically substantial distinction between the two groups with respect to mean age, gender, smoking status, comorbidities, complete blood count (CBC), laboratory parameters, or echocardiographic findings.

### 2.1. Change in Biochemical Parameters, Cardiac Markers, and Non-Coding RNA between Both Groups

The actually imply vitamin D level in Group 2 was significantly higher than in Group 1, however there wasn’t any other substantial difference in the laboratory parameters between the two groups, including CBC parameters, renal function parameters, or hepatic function parameters. There was a substantial difference in cardiac indicators between the two groups, favoring Group 2 who received vitamin D treatment including PIIINP and soluble ST2 as presented in Table 2. Additionally, the levels of mir361-5p, lnc-MEG3, and lnc-Chaer were considerably greater in Group 1 than in Group 2, in both groups. On the contrary, there was no discernible difference between both groups regarding mir675 and lnc-H19 as shown in Table 2.

### 2.2. Change in Echocardiographic Findings and HF and MACE Occurrence between Both Groups

The echocardiographic parameters considerably varied between the two groups, with LVEF being significantly higher in Group 2 than in Group 1 (*p* = 1.1 × 10^−4^ *) and LVESV and LVEDV being markedly decreased in Group 2 than in Group 1 (*p* = 0.0075 and 0.002, respectively) (Table 2). In terms of the prevalence of HF and MACE, the percentage of patients in Group 1 was substantially higher than Group 2 (*p* = 0.001 and 0.009, respectively). Additionally, as indicated in Table 2, Group 1 had a substantially larger percentage of patients who had more than one MACE compared to Group 2 (*p* = 0.043). While Table S2 and Figure 2 show how the levels of non-coding RNA and cardiac biomarkers changed in both groups among various genotypes.

### 2.3. Patterns of Prevalence of the Tested Polymorphisms in the Study Population and among Both Groups

All of the SNPs were analyzed to ensure that their genotype frequencies were in agreement with Hardy–Weinberg equilibrium (HWE), and as can be shown in Table 3, none of the evaluated polymorphisms showed any deviations from HWE. Furthermore, as shown in Table 4, there was no discernible difference in the frequency of genotypes between the two groups. patients who experienced heart failure had a greater frequency of the mutant AA genotype for the *Taq I rs731236* variants compared to patients who did not develop heart failure (*p* = 4.2 × 10^−7^). According to Table 5, there was no statistically significant difference in the genotype frequencies between the heart failure and non-heart failure groups for any of the other three SNPs evaluated.

Patients who experienced MACE had a significantly lower frequency of the mutant TT genotype for *Bsm I rs1544410* variations compared to the non-MACE group (*p* = 4.8 × 10^−4^). In addition, the mutant GG genotype was considerably more common in patients who suffered MACE (*p* = 0.003) for *Fok I rs2228570*. Table 6 shows that there was no statistically significant difference in the frequency of genotypes between the MACE and non-MACE groups for the other two SNPs evaluated. Table 7 further shows that the mutant TT genotype for *Bsm I rs1544410* was considerably lower among individuals who experienced several MACEs than among those who experienced only a single incident.

### 2.4. Impact of Tested Single-Nucleotide Polymorphisms on Susceptibility to Heart Failure and MACE

*Taq I rs731236* was significantly associated with heart failure after adjusting for multiple comparisons, and those with AA mutant genotypes had a markedly elevated risk of developing heart failure, as shown in Table 8. Moreover, individuals who carried the wild form of the *Bsm I rs1544410* genotype were at much higher risk of developing MACE than those who carried the mutant form; however, there was a considerable reduction in MACE risk among those with the *Fok I rs2228570* AA wild genotype. In addition, patients whose *Bsm I* alleles were mutant for the rs1544410 variant had a far lower probability of developing multiple MACE than did those whose alleles were wild as shown in Table 8.

### 2.5. Correlation Analysis among Cardiac Markers and Non-Coding RNA Serum Levels (Pearson Correlation)

**Figure 3:**
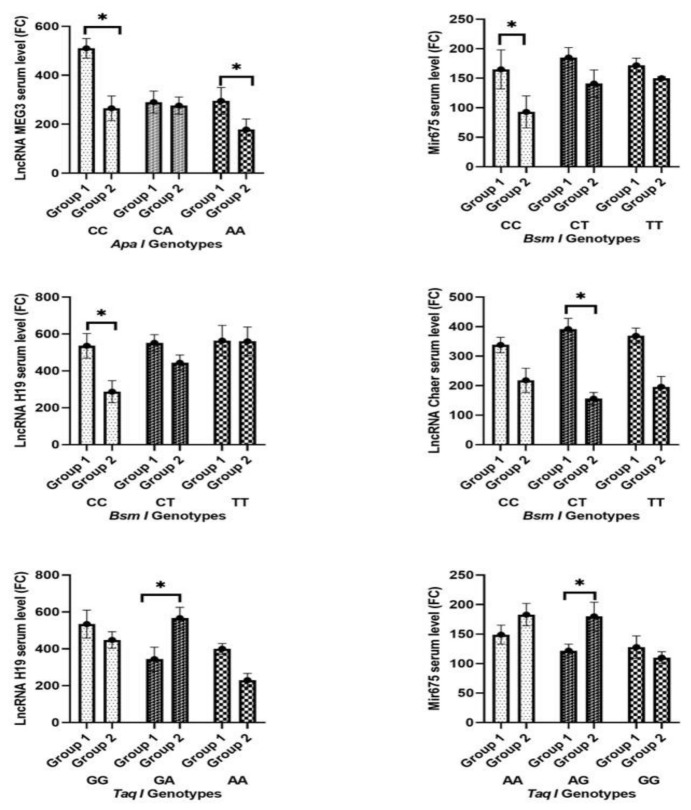
Change in cardiac biomarkers and non-coding RNA levels in both groups among different genotypes. * Significant difference (*p* < 0.0125).

Correlation analysis in heart failure patients showed a significant favorable correlation between lnc-MEG3 and lnc-Chaer and a good positive correlation between mir361-5p and lnc-MEG3; however, a negative connection was seen between serum mir675 and mir361-5p, lnc-MEG3, and lnc-Chaer (Table 9). For MACE patients, a positive association was found between soluble ST2 and mir361-5p, as well as between serum mir675 and lnc-H19. A strong positive link was also discovered between mir361-5p and lnc-MEG3 and lnc-Chaer, as well as between lnc-MEG3 and lnc-Chaer. Alternatively, a negative association was discovered between soluble ST2 and lnc-Chaer, and between mir675 and lnc-MEG3 and lnc-Chaer (Table 10). The Change in cardiac markers and non- coding RNA levels among different genotypes in both groups was compared and presented in Figure 3.

### 2.6. ROC Curve Analysis

The ROC analysis showed that among the evaluated cardiac biomarkers and non-coding RNAs between cases of heart failure and those without heart failure, serum lncRNA MEG3 demonstrated the highest AUC (0.92), specificity (96.7%), and sensitivity (87.2%), followed by mir361showing AUC (0.913), specificity (100%), sensitivity (60%), and finally lncRNA-Chaer the AUC was (0.852), the specificity (100%), and the sensitivity (70.8%). The area under the curve (AUC) for mir675 was 0.816%, while its specificity was 86.70% and its sensitivity was 71.4%. For cardiac biomarkers for heart failure prediction, PIIINP has the highest area under the curve (0.892), specificity (97.2%), and sensitivity (85.3%), whereas soluble ST2 comes in second with AUC (0.829), specificity (87.2%), and sensitivity (86%).

Serum mir 361 and lncRNA MEG3 showed the highest AUC (0.877), specificity (96.8% and 90.3%, respectively), and sensitivity (65.1% and 84.5, respectively) among the evaluated cardiac biomarkers and non-coding RNAs for differentiation between MACE and non-MACE cases, while lncRNA-Chaer showed AUC (0.802), specificity (96.8% and 90.3%, respectively), and sensitivity (72.5%).

**Figure 4:**
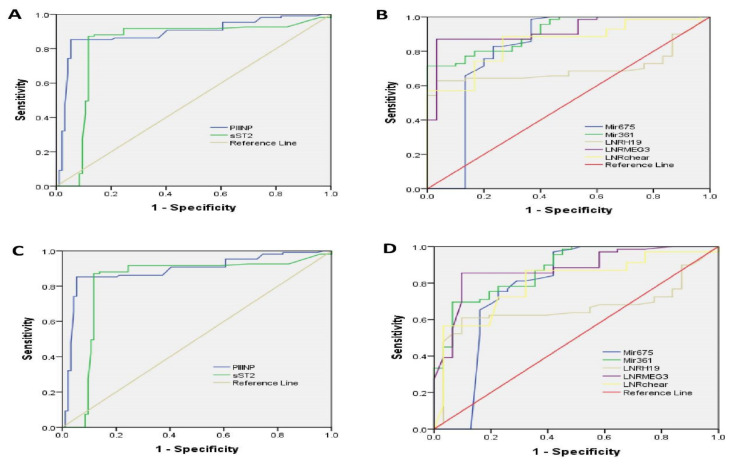
Receiver Operating Characteristic curves (ROC) for investigation of cardiac Bi-omarkers (**A**) and ncRNA (**B**) in heart failure predication among acute coronary syndrome (ACS) patients as well as of cardiac Biomarkers (**C**) and ncRNA (**D**) in major acute cardiovascular events (MACE) predication among ACS patients.

The area under the curve for mir675 was 0.787, while the sensitivity and specificity were 87.1% and 76.8%, respectively. lncRNA H19 had an AUC of (0.662), a specificity of 93.3%, and a sensitivity of 60.9%. For cardiac biomarkers for MACE prediction, PIIINP has the highest area under the curve (AUC) (0.866), specificity (93.0%), and sensitivity (82%) and is followed by soluble ST2 (AUC (0.801), specificity (87.8%), and sensitivity (77.5%) as shown in Figure 4, Table S3.

### 2.7. Multiple Regression Analysis to Detect Clinical and Genetic Predictor Variables of Vitamin D Response among ACS Patients

Multiple linear regression analyses were carried out to identify variability factors in changes in EF, PIIINP, soluble ST2, mir675, mir 371, lnc RNA H19, lnc RNA MEG3, and lnc RNA Chaer with vitamin D therapy. The results are presented in (Table 11 and Table 12).

## 3. Discussion

Over a billion people around the world suffer from vitamin D insufficiency, rendering it a real global health crisis. Calcitriol (1, 25(OH)2D) is the vitamin D’s active form D and is fully accountable for its many systemic effects, such as its anti-inflammatory, antithrombotic, and anti-atherosclerotic properties, which may help in avoid sudden cardiovascular ischemic episodes [15]. VDRs are essential for vitamin D functions since its active form, (1, 25(OH)2D), crosses the biological membranes and adheres to it, a complex is formed that connects with the retinoic acid receptor (RXR) to develop heterodimers. Specific genes’ transcription and repression are controlled by these heterodimers that bind to vitamin D response sites in their promoters [16]. The expression of more than a thousand genes and the epigenome are directly influenced by VDRs [17].

Vitamin D and vitamin D metabolites modulate VDR gene expression in a direct manner [18]. Previous studies conducted in the United States indicated that low vitamin D levels were related to lower VDR expression in mononuclear cells in peripheral blood [19]. VDR gene expression was also upregulated in white adipose tissue following vitamin D treatment of either 50, 1000, or 10,000 IU/kg for 3 months [20]. In addition, among Indian cancer patients with inadequate or deficient blood levels of vitamin D, an increase in VDR expression was seen in 110 cases of oral neoplasms, premalignant nodules, and oral cancer [21]. In addition, ACS pathophysiology includes the activation of inflammatory pathways, such as the leukotriene pathway, with subsequent reduction in several anti-inflammatory mediators, such as vaspin, and releases of multiple proinflammatory mediators that cause inflammatory changes and induce thrombosis via stimulation of platelets and coagulation factors, resulting in subsequent cardiac thrombosis [22,23]. Thus, maintain VDR gene expression by supplementation of ACS patients with vitamin D may be considered crucial for optimal management due to its anti-inflammatory and antiplatelets effects [15].

In this study, we investigated cardiac fibrosis markers and non-coding RNA blood levels in two groups: one that received conventional ACS treatment and the other that received standard ACS treatment with vitamin D supplementation based on vitamin D serum level. Moreover, we identified the genetic variants of vitamin D receptor (VDR) gene polymorphisms are linked to cardiac failure and MACE in ACS patients.

This study is the first to our knowledge to reveal the genetic variation and allele frequencies correlations of VDR genetic polymorphisms with heart failure and MACE among ACS patients in the Egyptian population. It was found that the *Taq I (rs731236)* mutant genetic variant significantly predicted the incidence of heart failure in patients with ACS. Among patients with ACS, the *Bsm I (rs1544410)* wild genetic variant was an excellent predictor of MACE, in contrast to the *Fok I (rs2228570)* wild genetic variant, which was associated with a reduced risk of MACE. The *Bsm I (rs1544410)* wild genetic variation was linked to multiple major adverse cardiac events (MACEs) across a 24-month observation period.

Vitamin D insufficiency was also found to be a predictor of MACE in a 2018 study by Navarro-Valverde and colleagues, which was consistent with our findings. The risk of major adverse cardiac events (MACE) was shown to be higher in patients with lower 25(OH)D levels (RR: 1.4; *p* = 0.037) compared to those with higher levels. Therefore, it was concluded that improving cardiovascular health and reducing the incidence of MACE might be achieved by normalizing vitamin D levels to >50 nmol/L with supplementation with calcifediol for 3 months [24,25].

Additionally, Vitamin D insufficiency was also linked to increased risk of ACS in a study conducted by Kamal and colleagues in 2022. Results from this cross-sectional investigation showed that 16.6% of ACS cases and 10% of controls had insufficient levels of vitamin D. 45% of those with ACS and 26% of those in the control group had insufficient vitamin D levels (*p* = 0.001). The study’s authors concluded that vitamin D supplementation should be suggested in the diagnosis and treatment of people with cardiovascular disease [26].

More so, an experimental study conducted by Jabeen et al. (2022) demonstrated that a single dose of vitamin D 200,000 IU had an immunomodulatory effect on various aspects of inflammation. Serum levels of C-reactive protein dropped after vitamin D supplementation in patients with non-ST-elevation acute coronary syndrome (*p* = 0.028 *), demonstrating its beneficial effects. As a result, when combined with standard ACS medications, vitamin D can have a profound effect on the inflammatory markers and lessen the likelihood of severe complications [27].

Inter-patient genetic variability has been related to drug response variability, potentially reducing the efficacy of treatment [28]. Calcitriol (1, 25(OH)2D) has been shown in numerous studies to interact with vitamin D receptors, which is thought to be the mechanism by which vitamin D exerts its effect (VDR). Therefore, it may be possible to better tailor Vitamin D supplements in patients experiencing ACS by identifying and understanding gene variants in VDR. Potentially, this could fill in the blank when it comes to the wide range of vitamin D’s effects on different people [13].

A significant correlation between the *Bsm I (rs1544410)* VDR polymorphism and vitamin D activity in coronary artery disease (CAD) patients following myocardial infarction was also found in a study by Raljevi and colleagues in 2021. Patients with coronary heart disease who experienced a cardiac event were genotyped for the VDR polymorphism *rs1544410*. Our findings are consistent with those of a previous study [29], which found that the T/T genotype of the *Bsm I (rs1544410)* VDR polymorphism is more common among myocardial infarction CAD patients.

Cardiovascular diseases (CVDs) continue to be the principal cause of mortality, and their poor prognosis persists despite the introduction of innovative therapeutic strategies. As a rapidly worsening CVD, ACS carries a high mortality rate. Clinical evaluations of ACS incidence currently rely on electrocardiography, catheterization, cardiac enzymes, and risk rating algorithms [30], but they each have their own set of drawbacks that must be considered. As it stands, cardiac troponin, the standard diagnostic marker, has a low specificity [31,32]. Angiograms are invasive, carry the possibility of complications, and are not useful for screening a large population of patients efficiently [33]. Therefore, it is crucial for patients with ACS to have accurate predictive and prognostic markers for myocardial injury so that clinicians can start treatment immediately.

Since, genome-wide screening methods have allowed for the discovery of previously unrecognized diagnostic or prognostic markers, as well as the discovery of potential new therapeutic targets. Subsequent studies have uncovered several lncRNA-based biomarkers for the detection of various cancers [34,35,36], but in the case of cardiovascular diseases, no such clinical trials were ever conducted. In the current study, we aimed to investigate the role of cardiac biomarkers PIIINP and soluble ST2 as well as non-coding RNAs mir675, mir361-5p, LNRH19, and LNRMEG3 in the development of heart failure and MACE in patients with ACS. In this context, susceptibility to heart failure and MACE was found to be significantly correlated with higher serum expressions of mir361-5p, LNRMEG3, and LNRchaer. Patients with ACS and MACE had lower protein expressions of mir675 and LNRH19. As far as the authors are aware, this is the first study to evaluate the aforementioned epigenetic markers as a consequence of the intake of vitamin D among ACS patients and its connection to the occurrence of HF and MACE.

Oxygen deprivation plays a major role in myocardial infarction (MI) by causing a high rate of cell death (apoptosis and necrosis) in viable cardiomyocytes. Involvement of lncRNAs as regulators in MI-induced apoptotic cell death has been the subject of several recent studies [37,38]. MI was associated with downregulation of several lncRNAs, including cardiac apoptosis-related lncRNA (Carl) [37] and mitochondrial dynamic-related lncRNA (Mdrl) [28]. Smaller infarct sizes were observed in vivo after adenoviral overexpression of Carl or Mdrl, both of which suppressed mitochondrial fission and cardiomyocyte apoptosis by downregulating pro-apoptotic miR-361 [14,39]. Consistent with previous research, we found that mir361 serum levels were raised in patients who had HF and MACE, and that these levels were favorably connected with LNRMEG3 and LNRchaer, and negatively correlated with LNRH19, however, those who received Vitamin D supplements had a significantly lower serum level.

As mentioned above, in vivo modulation of cardiac-specific lncRNAs may offer new targets for the treatment of CVDs, as they may improve cardiac function or slow pathological progression in the diseased heart. The ability to regulate LncRNA expression in vivo is crucial if these molecules are to be used as therapeutic targets [40,41].

Consistently with our results, Wu et al., observed that the expression of Meg3 was gradually elevated in the heart of wounded mice following MI. Additional gain-of-function and loss-of-function studies in rodent cardiomyocytes demonstrated pro-apoptotic roles for Meg3 [42]. Meg3 was also engaged in apoptosis control via its direct association with RNA-binding protein FUS, and it was directly increased by p53 in hypoxic situation (fused in sarcoma) [43]. Following the intramyocardial injection of an adeno-associated virus serotype 9 (AAV9) system containing Meg3 shRNA, cardiac function was significantly enhanced in adult mice with MI. Additionally, MEG3 demonstrated a strongly counter effect in cardiomyocytes generated from human stem cells and was upregulated in clinical heart failure samples [42].

In addition, the growth of Ang-II-stimulated cardiomyocytes may be suppressed by silencing Lnc-MEG3 or by overexpressing Lnc-Chaer [44,45,46]. Reduced phenylephrine-induced cardiomyocyte enlargement may result from either upregulation of Lnc-H19, uc.323, or Lnc-SNHG1, or downregulation of Lnc-Chast and Lnc-Ahit [47,48,49].

In contrast to our results, a prior investigation by Li and colleagues using pigs to evaluate lncRNA H19 showed that its expression was really elevated in two mice models of abdominal aortic aneurysm using a low-density lipoprotein receptor mutant pig model [50]. In addition, a recent study highlights the translational potential of lncRNA H19 by showing that the expression of this gene is suppressed in response to afterload augmentation in a pig model of left ventricular hypertrophy, in human cardiac tissues from patients with heart disease, in human stem cell-derived cardiomyocytes, and in sentient heart tissue [50,51]. H19 knock-out mice had worse cardiac hypertrophy after pressure overload [52].

At last, lnc-Chaer reduces the severity of cardiac hypertrophy by restricting polycomb repressive complex 2 (PRC2) activity to Inhibit histone H3 lysine 27 methylation (H3K27Me) in the promoters of genes in aberrant gene transcription owing to stress. If lnc-Chaer were overexpressed, PRC2 binding to the Myh7 and Acta1 genes would be reduced, leading to an increase in the expression of these hypertrophic genes and, ultimately, to hypertrophy [53]. Hypertrophy of the myocardium caused by excessive pressure loading might be prevented in the lnc-Chaer KO model, as shown below. Accordingly, the lncRNAs help keep the fetal contractile isoforms encoded by the hypertrophic genes expressed normally. The transcription of MYH7 and other associated genes that cause a hypertrophic morphology may occur when the level of certain lncRNAs changes in response to pathogenic stimulations of cardiomyocytes [53,54]. These results are consistent with those of our investigation, which revealed a large rise in lnc-Chaer serum levels in patients who experienced cardiac failure and MACE.

The study of the molecular mechanisms of cardiac hypertrophy has made significant strides during the past several decades. However, substantial challenges remain in identifying the pathogenic mechanisms behind cardiac hypertrophy in order to develop more effective therapies [55,56]. Numerous long non-coding RNAs undergo dynamic regulation throughout the beginning and progression of cardiovascular diseases. Many can act as novel classes of circulating biomarkers and/or perform crucial biological roles. Even if the silencing or overexpression methodologies still need further refining, our in vivo results, along with others, show that regulation of lncRNAs offers a viable new therapeutic option to treat CVDs. However, lncRNAs are anticipated to become significant additional strategies for the management of numerous diseases, including CVDs., in the near future [14,57].

## 4. Materials and Methods

### 4.1. Study Design and Patients

The current study was a prospective randomized controlled trial that was carried out at the Cardiology Department of Ain Shams University Hospitals (ASUH). all subjects executed a written consent agreement to participate in the study, which was authorized by the October 6th University’s ethics committee (approval number: PRC-Ph-2210037). Patients were considered for enrollment between July 2020 and October 2022; those with ST-segment elevation Acute Coronary Syndrome (ACS) with a 24 h onset and resting cardiac ischemic symptoms lasting 10 min and at least 2 of the following: ST-segment elevation 1 mm in 2 contiguous leads (not known to be preexisting or due to a coexisting disorder) or new LBBB with primary planned PCI; Positive biomarker demonstrating myocardial damage (Troponin I or T or CK-MB above the upper limit of normal); one of the following: age of 60 or older or history of myocardial infarction (MI) or coronary artery bypass grafting (CABG) or coronary artery disease (CAD) with less than 50% lumen diameter loss in at least two vessels or history of ischemic stroke, TIA, carotid stenosis (>50%), or cerebral revascularization or Insulin resistance and diabetes or chronic kidney failure [58]. Heart failure patients with a left ventricular ejection fraction (LVEF) of less than 40%, patients with inflammatory diseases, valvular diseases that were characterized as moderate to severe valvular stenosis or severe valvular regurgitation [grade III/IV], with recent valvular replacement in the past three months were excluded from the study.

Patients were divided into two groups at random in a ratio of 1:1using sequentially numbered, opaque-sealed envelopes to hide allocation [59]. A computer-generated randomization list was used to carry out the randomization. An independent statistician designed the randomization sequence. The allocation of treatments was disclosed to both patients and physicians.

Group 1 comprised of 125 ACS patients who were given ACS standard therapy alone without testing for vitamin D status, while Group 2 included 125 ACS patients who were given ACS standard therapy PLUS vitamin D based on serum vitamin D level (50,000 IU/Week for 8 weeks followed by 10,000 IU/Week for 4 months if serum level of 1, 25 dihydroxy vitamin D was 12 ng/mL) or (10,000 IU/Week for 6 months if serum level of 1, 25 dihydroxy vitamin D was >12 ng/mL).

The standard therapy for all ACS patients includes antiplatelet therapy with and aspirin 300 mg and ticagrelor 90 mg or clopidogrel 75 mg, it was up to the cardiologist’s decision. Patients undergoing thrombolysis do not receive ticagrelor [60,61]. Patients were given morphine or fentanyl for pain management, oxygen in case of hypoxia, and sublingual nitroglycerin for pain relief. It is important to note that the duration of dual antiplatelet therapy (DAPT) with aspirin and either clopidogrel or ticagrelor following PCI was 6 months then followed by long life aspirin [61]. Unless there were contraindications, patients were started on beta-blockers, statins, and ACE inhibitors as soon as possible [61].

### 4.2. Procedures

Patients who fulfilled the inclusion criteria had a full laboratory and echocardiographic assessment at baseline. Clinical and medical data such age, gender, weight, smoking habits, past medical history, and treatments, were collected from patient interviews and medical records. Patients who agreed to a vitamin D screening had 5 mL of whole blood drawn for DNA extraction and genotyping, and 2 mL of serum drawn for determination of 1, 25 dihydroxy vitamin D levels.

The echocardiographic evaluation was repeated 6 months later, and a second serum sample (5 mL) was taken to assess vitamin D status, biochemical markers (such as PIIINP and soluble ST2), and epigenetic markers (such as micro RNAs (Mir675 and Mir361-5p)) and long non-coding RNAs (LnRNA H19, LnRNA MEG3, and LnRNA Chaer). After letting the samples clot, the sera were separated using centrifugation (3500 rpm for 20 min at 25 °C, divided into two aliquots, and frozen at 80 °C until needed for biochemical testing.

The DHVD3 (1, 25-Dihydroxyvitamin D3) ELISA Kit catalog Number: ABCE-EL-0016 (Advanced Biochemicals, LLC. Lawrenceville, GE, USA) was used to calculate 1, 25 (OH) dihydroxy vitamin D3 levels. Human PIIINP was calculated using the N-Terminal Procollagen III Propeptide ELISA Kit catalogue number ABCE-EL-H0183 (Advanced BioChemicals, LLC. Lawrenceville, GE, USA) and human sST2 was calculated using the Human sST2 (soluble ST2) ELISA Kit catalogue number ABCE-EL-H1615 (Advanced BioChemicals, LLC. Lawrenceville, GE, USA).

Left ventricular ejection fraction (LVEF), which was evaluated using Simpson’s biplane rule, as well as left ventricular end-diastolic volume (LVEDV) and left ventricular end-systolic volume (LVESV), which were calculated from apical four chamber and apical two chamber imaging and left ventricular function, were all determined using two-dimensional (2D) transthoracic echocardiograms.

Patients were monitored for a total of 2 years to identify the occurrence of heart failure and secondary MACE, which is considered positive if patients were admitted to the intensive care unit due to experiencing a recurrence of cardiovascular events (recurrent ACS, ischemic stroke, coronary artery occlusion, and stent-related revascularization) while on dual antiplatelet therapy.

### 4.3. Serum Total RNA Extraction Including miRNAs and lncRNAs

Blood was drawn from every patient who had signed up, centrifuged at 1200× *g* for 15 min at 4 °C to separate the serum, and then stored at 80 °C before total RNA isolation. TRIzol reagent (Invitrogen; Thermo Fisher Scientific, Inc. Waltham, MA, USA) was used to extract total RNA in accordance with the manufacturer’s instructions. Purified RNA was reverse transcribed into cDNA using a PrimeScript RT Reagent Kit (Takara Biotechnology Co., Ltd., Dalian, China). Using a miScript SYBR Green PCR Kit (cat. no. 218073; Hilden, Nordrhein-Westfalen, Germany), the manufacturer’s protocol was followed to perform real-time PCR for the purpose of quantifying miRNA from cDNA. With the use of the housekeeping gene glyceraldehyde 3’-phosphate dehydrogenase (GAPDH) and an endogenous control for data normalization, the relative expression levels of miRNAs and lncRNAs (forward and reverse primers specified in the Table S1) were determined. Use of the 5 Plex Rotor Gene Real Time PCR Analyzer (Qiagen, Germany) was required for the amplification.

### 4.4. Genotyping Procedure

The peripheral blood leukocytes’ genomic DNA was extracted using a QIAcube apparatus (QIAGEN, Cairo, Egypt) according to the guidelines provided by the manufacturer. All four of the known polymorphisms *Apa I (rs7975232), Bsm I (rs1544410), Taq I (rs731236), and Fok I (rs2228570)* in the vitamin D receptor (VDR) gene were genotyped by use of the Taqman® analysis (Applied Biosystems, Foster City, CA, USA; assay ID: C 28977635 10, C 8716062 20, C 2404008 10, C 12060045 20) as per the manufacturer’s instructions.

### 4.5. Statistical Analysis

In this study, patients who were followed for at least six months after receiving vitamin D were evaluated for clinical response. Changes in LVEF, LVESV, LVEDV, and vitamin D status were the phenotypes of interest.

For the representation of continuous variables, the mean and standard deviation were utilized. Categorical variables were displayed as percentages and integers. With one degree of freedom, the chi-square test was used to examine Hardy–Weinberg equilibrium. The allele and genotype frequencies were compared using the relevant chi-square and Fischer’s exact tests. For each SNP, it was determined whether the tested VDR genotypes were associated with alterations in outcomes. A dominant model was tested on the four SNPs. An independent Student t-test was used to assess the changes in LVEF, LVESV, and LVEDV between Group 1 (those treated with vitamin D) and Group 2 (those not treated with vitamin D). The chi-square test was employed to compare the frequency of MACE and HF in each group.

The differences between genotype groups were examined using a one-way ANOVA. In a stepwise linear regression model, we included covariates with a *p*-value of less than 0.2. The association between the tested genotypes and the interesting outcomes was examined using the general linear model. Predictors of each variable measured were detected using multiple linear regression analysis. We included in the model co-variates with *p*-value < 0.02. Binary logistic regression was used to assess the correlation between the occurrence of MACE and HF.

*p*-values less than 0.05 were deemed significant for all published two-tailed *p* values. The Bonferroni-corrected significance level of *p* = 0.05 (4 tested SNPs) was used to determine which SNPs were significant, all statistical analyses were carried out with the software SPSS version 22.0 for Windows; SPSS Inc., Chicago, IL, USA.

## 5. Conclusions

The current study adds to the expanding evidence base that vitamin D supplementation in patients with acute coronary syndromes improves echocardiographic parameters by lowering markers of cardiac fibrosis such as pro-inflammatory cytokine (PINK)-1 and soluble somatostatin (soluble ST2). It was also found that the prevalence of the *Bsm I CC* wild, *Fok I GG*, and *Taq I AA* mutant genotypes of the VRD gene polymorphism might be associated with the occurrence of MACE and heart failure. As a result of these findings, vitamin D levelling was suggested as a supportive strategy in cardiovascular illnesses, and vitamin D therapy was added to routine ACS treatment in a safe manner.

## Figures and Tables

**Table 1:** Baseline Patients’ demographics and laboratory values in both groups.

Characteristics	Group 1 (*n* = 125)	Group 2 (*n* = 125)	(*p*-Value)
Age (yrs.); mean ± SD	52.58 ± 13.12	50.4 ± 10.37	0.47
Gender			
Male, *n* (%)	26 (20.8%)	29 (23.2%)	0.38
Female, *n* (%)	99 (79.2%)	96 (76.8%)	
Smokers, *n* (%)	61 (48.8%)	60 (48%)	0.493
Diabetes mellitus; *n* (%)	72 (57.6%)	74 (59.2%)	0.412
Hypertension; *n* (%)	88 (70.4%)	91 (72.8%)	0.318
Ever Smoked; *n* (%)	41 (32.8%)	43 (34.4%)	0.19
**CBC; mean ± SD**			
RBCs (10^6^ cells/µL)	4.57 ± 0.532	4.79 ± 0.586	0.105
Haemoglobin (gm/dL)	12.42 ± 1.787	13.00 ± 1.897	0.193
WBCs (10^3^ cells/µL)	10.00 ± 2.54	10.79 ± 2.78	0.126
Platelets (10^3^ cells/µL)	257 ± 69	280 ± 74	0.196
**Liver function Tests; mean ± SD**			
AST (U/L)	24.70 ± 10.63	29.58 ± 13.82	0.211
ALT (U/L)	25.88 ± 14.27	29.48 ± 11.08	0.318
**Kidney function Tests; mean ± SD**			
BUN (mg/dL)	19.85 ± 10.46	21.10 ± 12.60	0.667
Serum creatinine (mg/dL)	1.12 ± 0.32	1.10 ± 0.25	0.784
Serum K (mEq/L); mean ± SD	3.69 ± 0.29	3.57 ± 0.25	0.09
**Baseline echocardiographic parameters**			
EF (%)	59.83 ± 6.92	61.90 ± 6.05	0.16
ESV (mL)	75.33 ± 5.2	67.28 ± 4.1	0.18
EDV (mL)	106.73 ± 6.53	114.18 ± 8.21	0.204
**Comorbid diseases**			
Hypertension; *n* (%)	67 (53.6%)	73 (58.4%)	0.375
Diabetes; *n* (%)	52 (41.6%)	59 (47.2%)	0.248
Hyperlipidemia; *n* (%)	45 (36%)	63 (50.4%)	0.102

Statistical analysis: Values are means ± SD; Significance is at *p* < 0.05. SD—standard deviation; NSTEMI—non-ST segment elevation myocardial infarction; STEMI—ST segment elevation myocardial infarction; CBC—complete blood count; RBCs—Red blood cells; WBCs—White blood cells; AST—aspartate aminotransferase; ALT—alanine aminotransferase; BUN—blood urea nitrogen; K—potassium; EF—ejection fraction; ESV—end systolic volume; EDV—end diastolic volume; PIIINP—Procollagen type III N-Terminal Peptide.

**Table 2:** Patients’ laboratory values and Echocardiographic parameters after 6 months of vitamin D treatment in both groups.

Characteristics	Group 1 (*n* = 125)	Group 2 (*n* = 125)	(*p*-Value)
**CBC; mean ± SD**			
RBCs (10^6^ cells/µL)	4.6 ± 0.5	4.8 ± 0.4	0.153
Hemoglobin (gm/dL)	12.5 ± 1.7	12.9 ± 1.6	0.342
WBCs (10^3^ cells/µL)	10 ± 2.3	10.7 ± 2.1	0.154
Platelets (10^3^ cells/µL)	263 ± 58	283 ± 81	0.25
**Liver function Tests; mean ± SD**			
AST (U/L)	20.2 ± 9.4	22.5 ± 10.8	0.285
ALT (U/L)	20.4 ± 6.5	23.4 ± 9.1	0.105
**Kidney function Tests; mean ± SD**			
BUN (mg/dL)	18.2 ± 6.7	18.3 ± 9.8	0.972
Serum creatinine (mg/dL)	1.1 ± 0.2	1.2 ± 0.19	0.606
Serum K (mEq/L); mean ± SD	4.6 ± 0.5	4.8 ± 0.3	0.18
**Echocardiographic parameters after 6 months**			
EF (%)	36.4 ± 8.1	66.8 ± 5.9	1.1 × 10^−4^ *
ESV (mL)	90.5 ± 25.6	74.4 ± 16.2	0.0075 *
EDV (mL)	141.2 ± 16.6	122.8 ± 9.5	0.002 *
**Cardiac fibrosis marker; mean ± SD**			
PIIINP (ng/mL)	12.6 ± 3.8	4.7 ± 2.2	0.007 *
Soluble ST2 (ng/mL)	63.6 ± 10.6	25.7 ± 8.4	0.0004 *
Mir675	118.9 ± 28.2	153.7 ± 20.7	0.422
Mir361-5p	273.8 ± 40.6	139.1 ± 56.1	2.9 × 10^−4^ *
Lnc-H19	263.4 ± 45.2	319.7 ± 23.9	0.165
Lnc-MEG3	492.5 ± 55.8	226.2 ± 64.2	2.2 × 10^−6^ *
Lnc-Chaer	334.6 ± 27.8	200.5 ± 38.9	1.2 × 10^−5^ *
**Vitamin D status**			
Vitamin D level (ng/mL)	8.5 ± 2.6	31.4 ± 4.3	0.0008 *
**Clinical Outcomes**			
Heart Failure occurrence, *n* (%)	55 (44%)	30 (24%)	0.001 *
MACE occurrence, *n* (%)	56 (44.8%)	34 (27.2%)	0.009 *
**Number of MACE** One event, *n* (%) Two events or more, *n* (%)	43 (34.4%) 13 (10.4%)	29 (23.2%) 5 (4%)	0.043 *

Statistical analysis: Values are means ± SD; Significance is at *p* < 0.05. SD—standard deviation; CBC—complete blood count; RBCs—Red blood cells; WBCs—White blood cells; AST—aspartate aminotransferase; ALT—alanine aminotransferase; BUN—blood urea nitrogen; K—potassium; EF—ejection fraction; ESV—end systolic volume; EDV—end diastolic volume; Questionnaire; PIIINP—Procollagen type III N-Terminal Peptide; MACE—major acute cardiovascular event. *: significant difference (*p* < 0.05).

**Table 3:** Distribution of the examined genetic polymorphisms in the study population.

SNP	Minor Allele Frequency	Genotype	Number of Patients	Genotype Frequency	HWE ‡
		CC	93	37.60%	
***Apa I***		CA	119	47.60%	
**rs7975232**	0.4	AA	36	14.80%	0.96
		CC	88	35.20%	
***Bsm I***		CT	122	48.80%	
**rs1544410**	0.37	TT	38	16%	0.9
		GG	94	37.60%	
***Taq I***		GA	110	44%	
**rs731236**	0.42	AA	45	18%	0.74
		AA	85	34%	
***Fok I***		AG	123	49.20%	
**rs2228570**	0.33	GG	41	16.80%	0.31

‡ HWE *p*-value matches to the chi-square goodness-of-fit test findings, presuming one degree of freedom. SNP–Single Nucleotide Polymorphism; ‡ HWE, Hardy–Weinberg equilibrium.

**Table 4:** Distribution of VDR gene genotypes among both groups.

*Group*	*Apa I* rs7975232			*Bsm I* rs1544410			*Taq I * rs731236			*Fok I* rs2228570		
	CC *n* (%)	CA *n* (%)	AA *n* (%)	CC *n* (%)	CT *n* (%)	TT *n* (%)	GG *n* (%)	GA *n* (%)	AA *n* (%)	AA *n* (%)	AG *n* (%)	GG *n* (%)
***Group 1***	49 (39)	59 (47)	17 (14)	47 (38)	60 (48)	17 (14)	46 (37)	57 (46)	21 (17)	42 (34)	62 (49)	21 (17)
***Group 2***	44 (33)	60 (50)	21 (17)	41 (34)	62 (49)	21 (17)	48 (38)	53 (42)	24 (20)	43 (34)	61 (49)	20 (17)
***Statistics***	χ^2^: 1.26, *p* = 0.53			χ^2^: 0.86, *p* = 0.65			χ^2^: 0.38, *p* = 0.73			χ^2^: 0.09, *p* = 0.89		

Rs: referred sequence, *n* (%): number (percentage), χ^2^: Pearson Chi-Square.

**Table 5:** Distribution of VDR gene genotypes among Heart failure and non-heart failure groups.

*Group*	*Apa I* rs7975232			*Bsm I* rs1544410			*Taq I* rs731236			*Fok I* rs2228570		
	CC *n* (%)	CA *n* (%)	AA *n* (%)	CC *n* (%)	CT *n* (%)	TT *n* (%)	GG *n* (%)	GA *n* (%)	AA *n* (%)	AA *n* (%)	AG *n* (%)	GG *n* (%)
***No Heart Failure***	65 (40)	75 (46)	24 (14)	60 (37)	80 (49)	23 (14)	75 (46)	74 (45)	15 (9)	51 (31)	86 (53)	26 (16)
***Heart Failure***	28 (33)	44 (52)	21 (15)	28 (33)	42 (49)	15 (18)	19 (22)	36 (42)	30 (36)	34 (40)	37 (43)	15 (17)
***Statistics***	χ^2^: 1.11, *p* = 0.58			χ^2^: 0.73, *p* = 0.71			χ^2^: 29.4, *p* = 4.2 × 10^−7^ *			χ^2^: 2.28, *p* = 0.32		

Rs: referred sequence, *n* (%): number (percentage), χ^2^: Pearson Chi-Square, *: significant difference (*p* < 0.0125).

**Table 6:** Distribution of VDR gene genotypes among MACE and no MACE groups.

*Group*	*Apa I* rs7975232			*Bsm I* rs1544410			*Taq I* rs731236			*Fok I* rs2228570		
	CC *n* (%)	CA *n* (%)	AA *n* (%)	CC *n* (%)	CT *n* (%)	TT *n* (%)	GG *n* (%)	GA *n* (%)	AA *n* (%)	AA *n* (%)	AG *n* (%)	GG *n* (%)
***No MACE***	63 (40)	77 (49)	18 (11)	14 (9)	111 (70)	33 (21)	46 (37)	57 (46)	21 (17)	62 (39)	77 (48)	20 (13)
***MACE***	30 (33)	42 (47)	18 (20)	74 (82)	11 (12)	5 (6)	48 (38)	53 (42)	24 (20)	23 (25)	44 (49)	23 (26)
***Statistics***	χ^2^: 3.63, *p* = 0.16			χ^2^: 1.52, *p* = 4.8 × 10^−4^ *			χ^2^: 0.38, *p* = 0.73			χ^2^: 7.1, *p* = 0.003 *		

Rs: referred sequence, *n* (%): number (percentage), χ^2^: Pearson Chi-Square, *: significant difference (*p* < 0.0125).

**Table 7:** Distribution of VDR gene genotypes among one MACE event and more than one MACE groups.

*Group*	*Apa I* rs7975232			*Bsm I* rs1544410			*Taq I* rs731236			*Fok I* rs2228570		
	CC *n* (%)	CA *n* (%)	AA *n* (%)	CC *n* (%)	CT *n* (%)	TT *n* (%)	GG *n* (%)	GA *n* (%)	AA *n* (%)	AA *n* (%)	AG *n* (%)	GG *n* (%)
***One MACE***	21 (29)	38 (53)	13 (18)	35 (49)	21 (29)	16 (22)	21 (29)	34 (47)	17 (24)	20 (28)	35 (49)	17 (24)
***More than one MACE***	9 (50)	4 (22)	5 (28)	11 (61)	6 (33)	1 (6)	8 (44)	5 (28)	5 (28)	3 (17)	11 (61)	4 (22)
***Statistics***	χ^2^: 5.43, *p* = 0.067			χ^2^: 6.8, *p* = 0.002 *			χ^2^: 2.42, *p* = 0.301			χ^2^: 1.15, *p* = 0.56		

Rs: referred sequence, *n* (%): number (percentage), χ^2^: Pearson Chi-Square, *: significant difference (*p* < 0.0125).

**Table 8:** Risk factors for heart failure and MACE occurrence by binary logistic regression analysis.

Risk Factor	β^0^	*p*-Value	Odd Ratio	95%CI for Exp(B)
**Heart Failure risk factor**				
*Taql mutant/wild*	−1.75	3.9 × 10^−4^	2.74	1.28–3.41
**MACE risk factor**				
*Bsm I wild/mutant*	−0.362	1.26 × 10^−4^	6.4	2.45–8.12
*Fok I wild/mutant*	1.01	0.001	0.67	0.52–0.94
**More than one MACE risk factor**				
*Bsm I mutant/wild*	−1.39	0.007	0.25	0.13–0.68

CI: confidence interval. For *Taq I*, GA and GG are coded as 0 while AA carriers are coded as 1. For *Bsm I* CT and TT are coded as 0 while CC carriers are coded as 1. For *Fok I* AG and GG are coded as 0 while AA carriers are coded as 1.

**Table 9:** Correlation between cardiac biomarkers and non-coding RNA among Heart failure patients.

	PIIINP (ng/mL)	sST2 (ng/mL)	Mir675 (FC)	Mir361-5p (FC)	Lnc-H19 (FC)	Lnc-MEG3 (FC)	Lnc-Chaer (FC)
**PIIINP** **(ng/mL)**		0.097	0.038	−0.062	0.066	−0.130	−0.150
		*p* = 0.373	*p* = 0.728	*p* = 0.572	*p* = 0.546	*p* = 0.233	*p* = 0.169
**sST2** **(ng/mL)**	0.097		−0.135	0.096	−0.098	−0.155	−0.118
	*p* = 0.373		*p* = 0.217	*p* = 0.378	*p* = 0.368	*p* = 0.155	*p* = 0.278
**Mir675** **(FC)**	0.038	−0.135		−0.338	0.146	−0.337	−0.303
	*p* = 0.728	*p* = 0.217		*p* = 0.001 *	*p* = 0.181	*p* = 0.002 *	0.005 *
**Mir361-5p** **(FC)**	−0.062	0.096	−0.338		0.111	0.717	0.728
	*p* = 0.572	*p* = 0.378	*p* = 0.001 *		*p* = 0.308	*p* = 8.1 × 10^−9^ *	*p* = 2.2 × 10^−8^ *
**Lnc-H19** **(FC)**	0.066	−0.098	0.146	0.111		0.078	0.019
	*p* = 0.546	*p* = 0.368	*p* = 0.181	*p* = 0.308		*p* = 0.477	*p* = 0.863
**Lnc-MEG3** **(FC)**	−0.130	−0.155	−0.337	0.717	0.078		0.764
	*p* = 0.233	*p* = 0.155	*p* = 0.002 *	*p* = 8.1 × 10^−9^ *	*p* = 0.477		*p* = 1.3 × 10^−10^ *
**Lnc-Chaer** **(FC)**	−0.150	−0.118	−0.303	0.728	0.019	0.764	
	*p* = 0.169	*p* = 0.278	*p* = 0.005 *	*p* = 2.2 × 10^−8^ *	*p* = 0.863	*p* = 1.3 × 10^−10^ *	

Data are given as *r.* Statistical analysis was carried out using the Pearson’s correlation analysis. * Significant difference at *p* < 0.05; * Significant difference at *p* ≤ 0.01. FC—fold change; ml—milliliter; *n*—number; ng—nanogram; *r*—Pearson’s correlation coefficient.

**Table 10:** Correlation between cardiac biomarkers and non-coding RNA among MACE patients.

	PIIINP (ng/mL)	sST2 (ng/mL)	Mir675 (FC)	Mir361-5p (FC)	Lnc-H19 (FC)	Lnc-MEG3 (FC)	Lnc-Chaer (FC)
**PIIINP** **(ng/mL)**		−0.079	−0.052	0.047	0.145	−0.039	0.078
		*p* = 0.459	*p* = 0.627	*p* = 0.658	*p* = 0.172	*p* = 0.715	*p* = 0.466
**sST2** **(ng/mL)**	−0.079		−0.160	0.311	−0.067	−0.114	−0.0211
	*p* = 0.459		*p* = 0.131	*p* = 0.003 *	*p* = 0.527	*p* = 0.285	*p* = 0.046 *
**Mir675** **(FC)**	−0.052	−0.160		−0.332	0.306	−0.312	−0.204
	*p* = 0.627	*p* = 0.131		*p* = 0.001 *	*p* = 0.003 *	*p* = 0.003 *	*p* = 0.054
**Mir361-5p** **(FC)**	0.047	0.311	−0.332		−0.166	0.458	0.344
	*p* = 0.658	*p* = 0.003 *	*p* = 0.001 *		*p* = 0.119	*p* = 5.7 × 10^−5^ *	*p* = 0.001 *
**Lnc-H19** **(FC)**	0.145	−0.067	0.306	−0.166		−0.203	−0.082
	*p* = 0.172	*p* = 0.527	*p* = 0.003 *	*p* = 0.119		*p* = 0.054	*p* = 0.442
**Lnc-MEG3** **(FC)**	−0.039	−0.114	−0.312	0.458	−0.203		0.419
	*p* = 0.715	0.285	*p* = 0.003 *	*p* = 5.7 × 10^−5^ *	*p* = 0.054		*p* = 3.9 × 10^−4^ *
**Lnc-Chaer** **(FC)**	0.078	−0.211 *	−0.204	0.344	−0.082	0.419	
	*p* = 0.466	*p* = 0.046 *	*p* = 0.054	*p* = 0.001 *	*p* = 0.442	*p* = 3.9 × 10^−4^ *	

Data are given as *r.* Statistical analysis was carried out using the Pearson’s correlation analysis. * Significant difference at *p* < 0.05; * Significant difference at *p* ≤ 0.01. FC—fold change; ml—milliliter; *n*—number; ng—nanogram; *r*—Pearson’s correlation coefficient.

**Table 11:** Genetic and clinical predictors of ejection fraction and cardiac fibrosis markers.

Predictors	Model 1 Change in EF		Model 2 PIIINP		Model 3 Soluble ST2	
	β (S.E)	*p*-Value	β (S.E)	*p*-Value	β (S.E)	*p*-Value
*Bsml*	3.98 (0.57)	0.0001 *	−0.58 (0.25)	0.023 *	------	------
*Fokl*	2.57 (0.55)	0.002 *	------	------	------	------
*Taql*	−3.4 (0.53)	0.0003 *	9.5 (3.6)	0.0002 *	------	------
Age	−0.47 (1.8)	0.19	−1.4 (0.24)	1.1 × 10^−4^	------	------
Baseline EDV	0.189 (0.26)	0.0002 *	------	------	------	------
Baseline ESV	−0.287 (0.34)	0.0001 *	------	------	------	------
PIIINP	------	------	------	------	55.5 (11.9)	0.006 *
Mir675	------	------	------	------	−1.25 (0.6)	0.037 *
Mir155	------	------	0.05 (0.02)	0.008 *	------	------
Heart failure	−12.6 (0.87)	0.0005 *	1.3 (0.38)	0.001 *	27.4 (14.2)	0.02 *
MACE	2.28 (0.85)	0.007 *	------	------	------	------
Hypertension	------	------	−0.74 (0.36)	0.045 *	------	------
Smoking	1.5 (0.74)	0.04 *	−20.6 (5.7)	0.006 *	------	------
Intercept	36.2	1.9 × 10^−5^	24.1	0.0001 *	47.4	0.01 *
R^2^	0.552		0.05		0.103	

*Apa I*, *Bsm I*, *Fok I* and *Taq I* genotype were used as predictors (coded as 0, 1 and 2). Clinical variables with *p*-value < 0.20 were included in the model. * Variables gained statistical significance (*p* < 0.05), were kept in the model.

**Table 12:** Predictors of mircro RNAs and non-coding RNAs levels.

Predictors	Model 4 Mir675		Model 5 Mir155		Model 6 LnRNA H19		Model 7 LnRNA MEG3		Model 8 LnRNA Chaer	
	β (S.E)	*p*-Value	β (S.E)	*p*-Value	β (S.E)	*p*-Value	β (S.E)	*p*-Value	β (S.E)	*p*-Value
Soluble ST2	−0.08 (0.04)	0.043 *	------	------	------	------	------	------	------	------
PIIINP	------	------	1.59 (0.48)	0.001 *	−15.6 (5.3)	0.004 *	------	------	−3.35 (1.7)	0.05
Mir675	------	------	−0.5 (0.02)	0.005 *	1.19 (0.18)	0.0004 *	−0.62 (0.02)	0.002 *	0.17 (0.06)	0.005 *
Mir155	------	------	------	------	6.1 (0.38)	0.0008 *	0.69 (0.042)	0.002 *	1.59 (0.173)	0.0001 *
LnRNA H19	0.6 (0.12)	0.001	0.05 (0.004)	0.007 *	------	------	------	------	−0.16 (0.015)	0.0007 *
LnRNA MEG3	−0.48 (0.04)	0.0003 *	0.46 (0.037)	0.0002 *	------	------	------	------	1.11 (0.14)	0.0006 *
LnRNA chaer	------	------	0.132 (0.014)	0.0001 *	−1.71 (0.13)	0.0005 *	0.148 (0.014)	0.0003 *	------	------
Sex	------	------	------	------	------	------	−12.2 (94.6)	0.009 *	------	------
Heart failure	------	------	−9.4 (3.4)	0.006 *	------	------	------	------	29.7 (7.1)	0.013 *
Hypertension	23.4 (2.3)	0.023 *	------	------	−19.6 (3.5)	0.007 *	------	------	------	------
Intercept	27.6	2.1 × 10^−4^	7.3	0.22	79.2	0.24	38.1	0.0004 *	−41.6	0.049 *
R^2^	0.322		0.926		0.466		0.92		0.89	

*Apa I*, *Bsm I*, *Fok I* and *Taq I* genotype were used as predictors (coded as 0, 1 and 2). Clinical variables with *p*-value < 0.20 were included in the model. * Variables that gained statistical significance (*p* < 0.05) were kept in the model.

## Data Availability

Available upon request.

## Supplementary Materials

The following supporting information can be downloaded at: https://www.mdpi.com/article/10.3390/ph16020262/s1, Table S1: Forward and reverse primers for the relative expression levels of miR-675, miR-361-5p, lncRNA H19, lncRNA MEG3 and lncRNA Chaer, Table S2: Comparison of Cardiac biomarkers and non-coding RNA levels between different VDR genotypes among both groups; 1 and 2, and Table S3. The ability of the assessed cardiac biomarkers and ncRNAs for predication of heart failure and MACE among complicated ACS patients.
