# Supplementary material for: Impact of Vitamin D Supplementation on the Clinical Outcomes and Epigenetic Markers in Patients with Acute Coronary Syndrome
Source: Pharmaceuticals (Basel). 2023 Feb 9;16(2):262. doi: 10.3390/ph16020262 (PMC9967129; doi:10.3390/ph16020262)
Supplement: Supplementary file 1 [file pharmaceuticals-16-00262-s001.zip › pharmaceuticals-2113569-supplementary.pdf]

Supporting Information

Impact of Vitamin D Supplementation on the Clinical Outcomes and Epigenetic Markers in Patients with Acute Coronary Syndrome

Neven Sarhan [1], Ahmed E. Abou Warda [2], Saud Alsahali [3] and Abdalla Salah Alanazi [4,5] *

[1] Clinical Pharmacy Department, Faculty of Pharmacy, Misr International University, Cairo, Egypt; nevine.mohamed@miuegypt.edu.eg

[2] Clinical Pharmacy Department, Faculty of Pharmacy, October 6 University, Giza, Egypt; ahmedessamabouwarda@gmail.com

[3] Department of Pharmacy practice, Unaizah College of Pharmacy, Qassim University, Qassim, Saudi Arabia; s.alsahali@gu.edu.sa

[4] Department of Clinical Pharmacy, College of Pharmacy, Jouf University, Sakaka, Saudi Arabia; **Asdalananzi@ju.edu.sa**

[5] Health Sciences Research Unit, Jouf University, Sakaka, Saudi Arabia; Asdalananzi@ju.edu.sa

* Correspondence: ; Asdalananzi@ju.edu.sa

Table S1. Forward and reverse primers for the relative expression levels of miR-675, miR- 361-5p, lncRNA H19, lncRNA MEG3 and lncRNA Chaer.

Non-Coding RNAs	Forward and reverse primers
MiR-675	Forward 5′-TGGTGCGGAGAGGGC-3′<br>Reverse: 5′- GAACATGTCTGCGTATCTC-3′
MiR-361-5p	Forward 5′- TCAGAATCTCCAGGGGT -3′<br>Reverse: 5′- GAACATGTCTGCGTATCTC -3′
LncRNA H19	Forward 5′-ATCGGTGCCTCAGCGTTCGG-3′<br>Reverse: 5′-CTGTCCTCGCCGTCACACCG-3
LncRNA MEG3	Forward 5′-CTGCCCATCTACACCTCACG-3′<br>Reverse: 5′-CTCTCCGCCGTCTGCGCTAGGGGCT-3′
LncRNA Chaer	Forward 5′- GAGCCAAAAACCAACAAGGA-3′<br>Reverse 5′- GGCCCAGCTACTGTGCTAAC -3′
G6PDH	Forward: 5′-AGCCACATCGCTCAGACAC-3′<br>Reverse: 5′-GCCCAATACGACCAAATCC-3′

Table S2. Comparison of Cardiac biomarkers and non-coding RNA levels between different VDR genotypes among both groups; 1 and 2

Group	***Apa I* rs7975232**			***Bsm I* rs1544410**			***Taq I* rs731236**			***Fok I* rs2228570**		
	**CC n (%)**	**CA n (%)**	**AA n (%)**	**CC n (%)**	**CT n (%)**	**TT n (%)**	**GG n (%)**	**GA n (%)**	**AA n (%)**	**AA n (%)**	**AG n (%)**	**GG n (%)**
**PIIINP**												
Group 1	7.1±11.2	5.8±1.4	7.3 ±0.8	6.6±1.2	5.8±0.7	6.2 ±1.2	6.2±3.4	6.4±3.3	5.8±1.2	12.1±0.7	13.1±1.4	12.6±1.1
Group 2	5.8±0.64	4.9±1.1	5.1 ±0.9	5.5±0.89	4.9±1.1	4.5 ±0.6	4.6±2.2	5.1±2.6	5.9±1.3	8.1±1.9	5.2±0.9	7.7±1.5
P-value	0.36	0.49	0.73	0.123	0.65	0.07	0.52	0.112	0.71	0.16	0.06	0.056
**Soluble ST2**												
Group 1	78±12	52±24	47±34	72±9.9	71±6.5	64±4.6	64±19.3	62±12.7	59±20.2	62±12	68±9	73±11
Group 2	29±13	43±18	27±29	67±7.3	68 ±9.9	60 ±11.2	25±2.9	27±4.5	21±6.9	52±10	31±13	27±8.5
P-value	0.001*	0.25	0.054	0.06	0.57	0.46	0.29	0.14	0.11	0.96	0.16	0.153
**Mir675**												
Group 1	149±22	122±42	110±29	165 ±33	185±17	172±12	161 ±12	125±18	97±22	149±16	122±11	128±19
Group 2	183±34	180±51	128±33	93 ±27	141 ±23	150±15	150 ±23	182±9	172±11	183±19	180±24	110±10
P-value	0.051	0.11	0.13	0.04*	0.13	0.27	0.85	0.36	0.146	0.31	0.031*	0.133
**Mir361**												
Group 1	154±21	151±43	163±31	147 ±17	149±31	183±26	167±29	148±14	141±24	154±12	151±10	163±11
Group 2	152±52	131±52	118±54	167 ±52	136±28	84±11	127 ±16	153±13	101±18	152±31	131±24	117±14
P-value	0.45	0.32	0.115	0.002*	0.34	0.72	0.85	0.24	0.18	0.45	0.48	0.84
**LncRNA H19**												
Group 1	396±35	349±24	332±40	536 ±67	552±45	564±83	534±76	344±85	400±29	369±33	349±27	332±45
Group 2	410±17	530±38	294±52	287 ±60	344±62	560 ±78	448 ±45	567±58	230±37	491±48	565±8	543±33
P-value	0.123	0.546	0.114	0.002*	0.34	0.56	0.56	0.029*	0.064	0.84	0.75	0.65
**LncRNA MEG3**												
Group 1	510±15	290±45	295±43	168±33	193±43	208±28	195±23	166±19	165±44	176±12	170±19	192±13
Group 2	265±41	276±36	178±32	292±51	132±35	131±34	132±17	188±28	275±39	210±10	167±27	175±24
P-value	0.008*	0.345	0.001*	0.009*	0.11	0.21	0.63	0.159	0.25	0.39	0.81	0.71
**LncRNA Chaer**												
Group 1	334±34	372±37	374±33	311±20	338±26	392±34	369±26	313±21	325±36	334±21	326±19	374±16
Group 2	294±52	311±43	284±41	308±31	218±41	156±51	196 ±35	231±24	304±18	294±13	211±22	173±12
P-value	0.23	0.362	0.167	0.73	0.54	0.003*	0.28	0.56	0.43	0.255	0.32	0.76

Table S3. The ability of the assessed cardiac biomarkers and ncRNAs for predication of heart failure and MACE among complicated ACS patients.

Marker	Cut-off	Specificity %	Sensitivity %	AUC	95% CI	P-value
**Heart Failure prediction**						
**Cardiac Biomarkers**						
**PIIINP (ng/ml)**	9.6	97.2%	85.3%	0.892	0.834-0.94	1.9E-5*
**Soluble ST2 (ng/ml)**	45	87.2%	86%	0.829	0.762-0.896	2.4E-4*
**Non-Coding RNA**						
**Mir675 (FC)**	305.2	86.7%	71.4%	0.816	0.694-0.937	6.1E-4
**Mir361 (FC)**	108.7	100%	70.8%	0.913	0.859-0.967	6.9E-3
**LncRNA H19 (FC)**	653.3	96.7%	65.7%	0.701	0.600-0.802	0.002*
**LncRNA MEG3 (FC)**	445.6	96.7%	87.15	0.920	0.863-0.976	3.4E-3*
**LncRNA Chaer (FC)**	687.2	88.6%	86.7%	0.852	0.778-0.927	2.6E-6*
**MACE prediction**						
**Cardiac Biomarkers**						
**PIIINP (ng/ml)**	9.2	93.1%	82%	0.866	0.814-0.918	1.4E-3*
**Soluble ST2 (ng/ml)**	53.5	87.8%	77.5%	0.801	0.730-0.871	0.002*
**Non-Coding RNA**						
**Mir675 (FC)**	251.1	87.1%	76.8%	0.787	0.664-0.910	4.6E-3*
**Mir361 (FC)**	217.4	96.8%	65.1%	0.877	0.805-0.948	1.9E-5*
**LncRNA H19 (FC)**	653.2	90.3%	60.9%	0.662	0.553-0.770	0.01*
**LncRNA MEG3 (FC)**	610	90.3%	84.5%	0.877	0.804-0.950	1.8E-4*
**LncRNA Chaer (FC)**	820.6	96.8%	72.5%	0.802	0.711-0.891	1.4E-3*

AUC; Area under the curve; CI: confidence interval; *, significance at p-value<0.05; PIIINP, procollagen type III N-terminal peptide; LNC RNA: long noncoding RNA; Mir; micro-RNA; ncRNA; noncoding RNA.
